# Lymph nodes’ evaluation in relation to colorectal cancer staging among African Americans

**DOI:** 10.1186/s12885-015-1946-x

**Published:** 2015-12-16

**Authors:** Hassan Ashktorab, Temitayo Ogundipe, Hassan Brim, Anahita Shahnazi, Adeyinka O. Laiyemo, Edward Lee, Babak Shokrani, Mehdi Nouraie

**Affiliations:** 1 Department of Medicine and Cancer Center, Howard University College of Medicine, 2041 Georgia Avenue, Washington, DC 20060 USA; 2 Department of Pathology, Howard University College of Medicine, Washington, DC USA

**Keywords:** LN, Colorectal cancer, African Americans

## Abstract

**Background:**

Lymph nodes’ examination in colorectal cancer (CRC) resection specimens is an important determinant that aids in the accuracy of CRC staging and treatment outcomes. Current guidelines call for the examination of at least 12 lymph nodes (LN) in resected specimens in order to establish accurate staging.

**Aim:**

To investigate lymph nodes’ examination protocol as it relates to accurate CRC staging.

**Methods:**

We reviewed 216 African American CRC patients from 1996–2013 who underwent CRC resection and met inclusion criteria for this study. The number of retrieved LNs, length of resected specimens, tumor grade, stage, location, size and histology were examined.

**Results:**

The cohort study was made of 49 % males, median age was 63 years and 45 % of patients were at stage III and IV. The median (IQR) number of examined LNs was 15 (10–22) and the rate of patients with more than 12 examined LNs was 64 %. There was a gradual increase in the percentage of patients with adequate number (>12) of examined LNs during the study period (from 60 % in 1996–2000 to 84 % in 2010–2013 period, *P* = 0.014). Adequate LNs resection was neither associated with shift of stage from II to III (*P* = 0.3) nor with the changes from stage IIIa to IIIc (*P* = 0.9). Metastatic LNs were observed in 8 % of samples with LNs (>12) vs. 13 % of samples with <12 examined LNs (*P* = 0.1). Patients that had pre-surgical treatment (chemotherapy and radiotherapy) before surgery had <12 LNs examined. There was also a trend of having more examined lymph nodes in large tumors.

**Conclusions:**

Our study shows that there has been an increase in the number of lymph nodes examined in CRC resections since the advent of the current quality initiative. However this increase does not seem to affect the stage or percentage of metastatic lymph nodes’ detection in CRC patients.

## Background

Colorectal cancer (CRC) is the third most common cancer and the second most common cause of cancer related deaths in both men and women in the United States [1–3]. In 2013, it accounted for 102,480 new cases and 50,090 deaths [4]. There are racial differences in CRC incidence and mortality, when compared to other races, African Americans (AAs) are disproportionately affected and have a greater burden from this disease [5].

In 2011, AAs had the highest incidence of CRC (Male: 62.3/100,000 persons and Female: 47.5/100,000 persons) when compared to Non-Hispanic Whites (Male: 49.6/100,000 persons and Female: 37.3/100,000 persons) [6]. They also have poorer outcomes and are more likely to die from CRC when compared to other races [3]. Several studies have shown that AAs are more likely to be diagnosed with advanced stage CRC and to have poorer survival rates after diagnosis compared to whites [7–9].

One of the parameters used for CRC staging is the evaluation of lymph nodes to determine if they are positive or negative for cancer. In patients with non-metastatic CRC, lymph nodes’ status is the strongest pathologic predictor of patient outcome [10]. Several studies have demonstrated increased survival in patients who had a high number of lymph nodes evaluated [11]. Although this association cannot be fully explained, it is thought to be due to the fact that patients who have more extensive LNs’ evaluation are at lower risk of being understaged. Understaged patients receive inadequate treatment leading to lower survival rates [11].

Adequate evaluation of LNs is important for therapeutic and prognostic reasons. Patients with positive LNs have to undergo adjuvant post-surgery chemotherapy because of the high risk of tumor recurrence [12]. This serves also to determine the accuracy of staging in patients with CRC [12]. The current standard of care recommended by the 1990 working party report to the World Congresses of Gastroenterology and also by the 2002 American Joint Committee on Cancer (AJCC) advocates for the examination of at least 12 lymph nodes in patients with CRC [13].

Our study aims to investigate the number of examined lymph nodes, their associated features and the pathology protocol used for their evaluation as these relate to proper and accurate staging of CRC in African Americans afflicted with this disease.

## Methods

### Patients

We reviewed the medical records of 216 African American patients with a diagnosis of CRC from January 1, 1996 to December 30, 2013. Data was retrieved from the database of the Department of Pathology and the Tumor registry unit of the Cancer Center at Howard University Hospital, an inner city tertiary institution in Washington DC. The Institutional Review Board at Howard University Ethics Committee approved the research protocol (06-MED-39). Due to the retrospective nature of the study no written informed consent for participation in the study was needed and no children were included in the study. The pathology reports on the tumor and specimen features as well as the examined and positive lymph nodes were investigated for each patient by GI pathologists (E.L. and B. S.).

### Pathological evaluation

Gastrointestinal Pathologists evaluated all specimens in this study. Colectomy specimens were evaluated based on grade, stage and other features. Foci of metastatic adenocarcinoma were explored. The foci usually locate in subcapsular specs and later involve parenchyma. During the gross evaluation of each resected specimen, all grossly negative or equivocal lymph nodes have been submitted entirely for microscopic evaluation. The accuracy and predictive value of stage assignment are directly proportional to the thoroughness of the surgical technique in removing all regional nodes and the pathologic examination of the resection specimens in identifying and harvesting all regional lymph nodes for microscopic assessment.

### Statistical analysis

Distribution of categorical variables was assessed by table of frequency and for continuous variables by median (interquartile range). We tested the relationship between the number of examined LNs (as a continuous variable or as two categories of >12 vs. ≤ 12) and stage by ANOVA or Chi-square.

## Results

### Clinicopathological features of the analyzed cohort

During the 17 years of review, we had a total of 216 CRC patients, 50 % of them were operated after 2007 (Table 1). The median age was 63 years old (interquartile range = 53-72) and 49 % (*n* = 106) of the study population was male. Ninety seven percent of tumors were adenocarcinoma and most were low grade tumors (95 %, *n* = 184). Most tumors were located at the ascending colon (41 %, *n* = 86) and rectosigmoid (37 %, *n* = 77).

**Table 1:** Distribution of demographic, clinical and pathologic CRC variables

	Number	Results
Age, median (Interquartile range)	214	63 (53–72)
Male, no (%)	216	106 (49)
Location	210	
Ascending		86 (41 %)
Transverse		21 (10 %)
Descending		26 (12 %)
Rectosigmoid		77 (37 %)
Histology	215	
Adenocarcinoma		179 (83 %)
Mucinous Adenocarcinoma		31 (14 %)
Other		5 (3 %)
Chemotherapy before surgery, no (%)	216	11 (5 %)
Radiotherapy before surgery, no (%)	216	11 (5 %)
Grade	193	
Low		184 (95 %)
High		10 (5 %)
Stage, no (%)	209	
I		46 (22 %)
II		71 (34 %)
III		74 (35 %)
IV		18 (9 %)
Number of LN resected	214	
≤12		77 (36 %)
>12		137 (64 %)
Median (interquartile)		15 (10–22)
Tumor size, Median (interquartile) cm	192	4.5 (3.0-5.7)
Specimen size, Median (interquartile) cm	188	33 (21–61)
Number of metastatic LNs, Median (interquartile)	214	0 (0–2)
Ratio of metastatic LNs to total LNs examined, Median (interquartile)	214	0 (0–0.09)
*Some of the data is missing		

*n* = 216*

### CRC stage distribution and lymph nodes’ examination

**Fig. 1:**
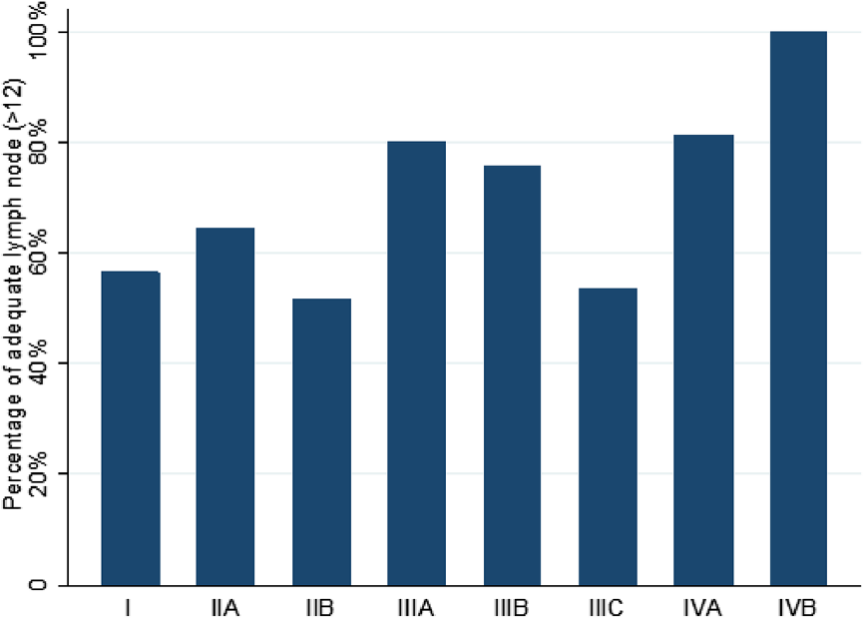
Percentage of adequate LNs examined at different stages of CRC in in African Americans. For stages IIIa, IIIb and IIIc, these percentages were 80 %, 76 % and 54 %, respectively

Thirty four percent of patients had stage II tumors [IIa(20 %), IIb(14 %)], 35 % had stage III [IIIa (2 %), IIIb (20 %), IIIc (13 %)], 9 % had stage IV disease[Iva (8 %), IVb (1 %)]. The median (IQR) number of examined LNs was 15 (10–22) and the rate of patients with more than 12 resected LNs was 64 %. Among patients with stage II, the frequency of adequate LNs resection was 59 % vs. 67 % in patients with stage III (*P* = 0.3). These frequencies at stage IIIa, IIIb and IIIc were 80 %, 76 % and 54 %, respectively (*P* = 0.1; Fig. 1). Metastatic LNs were observed in 8 % of samples with adequate number of examined LNs and in 13 % with less than 12 examined LNs (*P* = 0.1).

The median number of examined LNs in patients with stage I and II was 15 and 14, respectively. In patients with stage IIIa, the median number of examined LNs was 19, decreasing to 15 in patients with stage IIIb and 14 in patients with stage IIIc (P for trend = 0.2). The median number of lymph nodes in patients with stage IV was 17.

### Lymph nodes’ examination and other clinicopathological characteristics

Patients with inadequate number of examined lymph nodes were older (median age of 68 yrs vs. 61 yrs, *P* = 0.002). The frequency of adequate LN in 120 patients over 60 was 59 % (vs. 71 % in 92 patients <61 years old, *P* = 0.08). The proportion of metastatic lymph node was not different between age groups (*P* = 0.3). Presurgical radiotherapy and chemotherapy were more frequent in this group of patients (*P* = 0.05). Gender, tumor location or sizes of surgical specimen were not significantly different between these two groups. There was a gradual increase in the percentage of patients with adequate number (>12) of examined LNs during the study period (from 60 % in 1996–2000 to 84 % in 2010–2013 period, *P* = 0.014). The number of metastatic lymph nodes and also the ratio of metastatic to total lymph nodes were not different between the two groups (Table 2). Also, the number or ratio of metastatic lymph nodes were not correlated to tumor size (*P* = 0.1).The frequency of adequate lymph nodes or stage were not significantly different between pathologists. There was a significant correlation between tumor extent (T) and number of metastatic lymph nodes (*r* = 0.28, *P* = 0.0001).

**Table 2:** Clinical and pathologic characteristics of CRC by number of examined lymph nodes

	≤12 LNs *n* = 67-76	>12 LNs *n* = 121-136	*P* value
Age, median (Interquartile range)	68 (56–77)	61 (62–69)	0.002
Male, no (%)	42 (55)	63 (46)	0.2
Location			0.3
Ascending	28 (37 %)	56 (42 %)	
Transverse	9 (12 %)	12 (9 %)	
Descending	6 (8 %)	20 (15 %)	
Rectosigmoid	32 (43 %)	45 (34 %)	
Chemotherapy before surgery, no (%)	7 (9 %)	4 (3 %)	0.05
Radiotherapy before surgery, no (%)	7 (9 %)	4 (3 %)	0.05
Tumor largest size, Median (interquartile) cm	4.0 (3.0-5.5)	4.5 (3.0-5.7)	0.4
Specimen largest size, Median (interquartile) cm	31 (18–67)	33 (23–60)	0.9
Number of metastatic LNs, Median (interquartile)	0 (0–1)	0 (0–2)	0.1
Ratio of metastatic LNs to total LN examined, Median (interquartile)	0 (0–0.14)	0 (0–0.09)	0.6

### Subgroup analysis of patients with less than 12 lymph node examined

Sixty seven (31 %) patients had less than 12 LNs examined. In this subgroup of patients there was no correlation between the number of examined LNs and that of metastatic ones (*r* = 0.18, *P* = 0.1). There was also no correlation with stage (*r* = 0.2, *P* = 0.2). Also in this subgroup, increasing the number of examined lymph nodes did not shift the stage from II to III (*P* = 0.6).

## Discussion

Adequate examination of lymph nodes is important in patients with CRC; because lymph nodes’ status is a strong predictor of long term outcome in CRC patients [10]. Current guidelines recommend that at least 12 LNs should be harvested and examined in patients with CRC [14]. In this study among African American patients with CRC, we shows there has been an increase in the number of lymph nodes examined in colon cancer resection specimens since the advent of the current quality initiatives. The increase however does not lead to stage migration in our cohort of patients or to an increase in the percentage of metastatic lymph nodes.

The number of examined lymph nodes after colectomy is important for accurate staging and treatment design and also serves as a quality care indicator at the hospital level [15, 16]. Various factors influence the number of LNs retrieved including tumor’s size and stage [10], patient’s age and gender [17], surgeon and surgical procedure [18], pathologist [18] and presurgical chemotherapy/radiotherapy [19, 20]. Studies have shown that ethnic minorities are consistently underrepresented and underreported in medical research studies [21]. Cone et al. explored the relationship between lymph nodes’ count and ethnicity and indicated ethnicity as an important variable when assessing lymph nodes’ count with Hispanics having a lower chance of having ≥12 LNs evaluated when compared to Caucasians [18]. There are however no studies showing this relationship in African Americans. Our study was conducted in an African American urban patient population to address this gap of knowledge.

Our study showed that the majority (64 %) of patients in our cohort had more than 12 LNs examined with a median number of 15 LNs (range: 10–22). Also 84 % of CRC sample in recent years (2010–2013) had more than 12 LNs. This number exceeds the recommended benchmark of 12 LNs and is in compliance with the national guidelines. This result is not consistent with previous studies where the median number of examined LNs was less than 12 [10]. Our results are also not consistent with previous studies that show that African Americans are likely to undergo inadequate lymph nodes’ examination [22]. Our findings reflect that our procedure for the retrieval and examination of LNs are very aggressive and are way above the current gold standard. Of the 36 % of patients in our study with less than 12 examined LNs, 12 % had no further need for LNs’ examination since positive LNs for staging were already detected while in the remaining 24 % of patients with less than 12 examined LNs, no further LNs could be retrieved from the colectomy specimens.

In 2007, the National Quality Forum listed the presence of at least 12 lymph nodes in a surgical resection specimen among the key quality measures for colon cancer care in the United States [14]. Increasingly, however, evidence indicates that this bar should be raised, as the greater the number of examined LNs, the greater the likelihood of detection of metastasis, suggesting that no minimum number of LNs accurately or reliably stages all patients [23, 24]. Indeed, in a recent case, we examined 33 LNs to find only one positive after reviewing the first 24 LNs. In this case, checking just 12 or even 24 LNs would have resulted in missing a positive LN and affected the treatment regimen plan for this patient.

More importantly, it has been shown that clinical outcome is linked to lymph nodes retrieval in stage II disease. Numerous studies have shown that conventional pathologic examination of increased numbers of lymph nodes is itself associated with an increased survival advantage in stage II disease [15], indicating a positive effect of optimal mesenteric resection by the surgeon and optimal LNs retrieval and examination from the resection specimen by the pathologist.

As previously mentioned the number of lymph nodes recovered from resection specimen is dependent on several factors. Surgical technique, surgery volume, and patient factors (like age, size of tumor, size of specimen, administration of neoadjuvant therapy and anatomic variation) alter the actual number of nodes in a resection specimen, but the diligence and skill of the pathologist in identifying and harvesting lymph nodes in the resection specimen are also major factors. Lymph nodes may be more difficult to identify in specimens from elderly patients [25] and after neoadjuvant therapy [26] . In our study 56 % (*n* = 120) of our patients were elderly with 59 % having adequate number of LNs examined (vs. 71 % in 92 patients <61 years old, *P* = 0.08). This is consistent with other studies that report a negative correlation between lymph node yield and increasing age [27]. Because it has been shown that nodal metastasis in colorectal cancer is often found in small lymph nodes (<5 mm in diameter), diligent search for lymph nodes is required on gross examination of resection specimens.

Patients with stage III or IV colorectal cancer may receive presurgical chemotherapy and radiotherapy to reduce the number of metastatic LNs and to shrink the size of the tumor. In our study 11 (5 %) of our patients had presurgical treatment with the majority (*n* = 7) having less than 12 LNs examined (Table 2). This is consistent with other studies that showed reduced LNs yield in patients that received presurgical treatment [25, 26]. Another important factor is the size of tumor and size of specimen, increased specimen/tumor size positively correlated with higher number of LNs available in colectomy specimens, this difference was however not statistically significant in our cohort.

The decision to create the current guidelines was based on the assumption that the examination and analysis of more lymph nodes in patients with CRC will help prevent the omission of positive lymph nodes [27]. Based on the current American Joint Committee on Cancer (AJCC) TNM staging system, discovery of positive lymph nodes results in a shift in stage from stage I or II disease to stage III or IV. However, our study shows that increased examination and adequate lymph node retrieval was not associated with a shift in stage from stage II to III (*P* = 0.3). It was also not associated with a change in stage from stage IIIa to IIIb or IIIc. This is consistent with other studies showing that increased examination of lymph nodes is not associated with increase in node positive patients [11, 28, 29].

There was an increase in the percentage of patients who had more than 12 LNs examined as we upstage. Baxter et al. report tumor factors as important determinants of lymph nodes retrieval [10]. They also observed that more lymph nodes are examined in patients with stage II and III compared to patients with stage I [10]. This is consistent with our study in which there was a marked increase in the percentage of patients that had more than 12 LNs examined as we upstage. It was particularly noticeable in patients with stage IIIa and up. Also in patients with stage III disease, there was a steady decline in the number of examined LNs as we upstage from IIIa to IIIb and then IIIc. This may be attributed to the fact that in patients with stage III, positive LNs are important for staging and once the required numbers of positive nodes are detected, the pathologists may not aggressively look for other lymph nodes (*p* = 0.11, stage 3 *p* = 0.058).

Our study has some limitations. It is a retrospective study; it is therefore limited by differences in data input over time and not having the chance to review the cases again, and adherence with previous guideline. It is also a single institutional study with a relatively small sample size. We are going to eliminate this limitation in our prospective future study.

## Conclusion

Our study shows there has been an increase in the number of lymph nodes (>12) examined in colon cancer resection specimens since the advent of the current quality initiatives. The increase however does not lead to stage migration in our cohort of patients or to an increase in the percentage of metastatic lymph nodes. Metastatic LNs were observed in less of samples with LNs (>12) than samples with <12 LNs. There was also a trend of having more examined lymph nodes in large tumors.

## Abbreviations

CRC: colorectal cancer
LN: Lymph node
AA: African American
